# Shrinkage Behavior of Strength-Gradient Multilayered Zirconia Materials

**DOI:** 10.3390/ma18143217

**Published:** 2025-07-08

**Authors:** Andrea Coldea, John Meinen, Moritz Hoffmann, Adham Elsayed, Bogna Stawarczyk

**Affiliations:** Department of Prosthetic Dentistry, University Hospital, LMU Munich, 80336 Munich, Germany; john.meinen@med.uni-muenchen.de (J.M.); moritz.hoffmann@med.uni-muenchen.de (M.H.); adham.elsayed@med.uni-muenchen.de (A.E.); bogna.stawarczyk@med.uni-muenchen.de (B.S.)

**Keywords:** zirconia, multilayer, multigenerations, shrinkage

## Abstract

To investigate the sintering shrinkage behavior of multigeneration, multilayer zirconia materials using geometrical measurements. Seven zirconia CAD/CAM materials were analyzed, comprising two mono-generation zirconia (HTML: Katana Zr, HTML Plus, 3Y-TZP; UTML: Katana Zr, UTML, 5Y-TZP) and five strength-gradient multilayer zirconia (AIDI: optimill 3D PRO Zir; PRIT: Priti multidisc ZrO_2_ multicolor; UPCE: Explore Esthetic; ZCPC: IPS e.max ZirCAD Prime; ZYML: Katana YML) materials. Cubes (10 × 10 × 10 mm^3^) were milled in varying positions within the disks. Geometrical measurements were applied before and after dense sintering using a micrometer screw gauge, light microscopy, as well as surface scans and shrinkages were calculated. Data were analyzed using Kolmogorov–Smirnov, five-way ANOVA followed by the Scheffé post hoc test, and partial eta squared, as well as the Kruskal–Wallis test, including Bonferroni correction (*p* < 0.05). The highest influence on the shrinkage was exerted by the zirconia material (^η^_P_^2^ = 0.893, *p* < 0.001), followed by the test method (^η^_P_^2^ = 0.175, *p* < 0.001), while the vertical and horizontal position and measurement point showed no impact on the shrinkage results (*p* = 0.195–0.763) in the global analysis. Depending on the test method, the pooled shrinkage values of all tested zirconia materials varied between 17.7 and 20.2% for micrometer screw gauge, 17.7 and 20.1% for light microscopy, and 17.8 and 21.1% for surface scan measurements. The shrinkage values measured in the upper, middle, and lower multilayered vertical direction did not differ significantly in the global analysis for the multilayer materials. Therefore, a uniform shrinkage of these strength-gradient multilayer zirconia materials within clinically relevant restorations can be assumed.

## 1. Introduction

Pure zirconia lacks the mechanical properties required for reliable use in dental restorations. Through the incorporation of dopants such as alumina, yttria, magnesia, cerium, and ytterbia [1], and by applying specific sintering temperatures and procedures [2], zirconia can be stabilized in the tetragonal, tetragonal/cubic, or cubic phase. In dentistry, these modified materials are referred to as yttria-stabilized tetragonal zirconia polycrystals (Y-TZP). The most widely used variants include 3Y-TZP, 4Y-TZP, and 5Y-TZP. The 3Y-TZP contains 3 mol% Y_2_O_3_ and 0.05–0.25% Al_2_O_3_ and is partially stabilized in the metastable tetragonal phase. Under mechanical stress, it is capable of transforming into the monoclinic phase, contributing to transformation toughening [3]. By contrast, 4Y-TZP (4 mol% Y_2_O_3_, 0.05% Al_2_O_3_) is predominantly tetragonal with a minor cubic phase content, while 5Y-TZP contains up to 50% cubic phase (5 mol% Y_2_O_3_, 0.05% Al_2_O_3_) [4,5]. The precise composition of these additives plays a crucial role, as it directly impacts both the mechanical strength and optical properties, particularly translucency [6,7,8].

Y-TZP zirconia is widely applied in restorative dentistry, with indications ranging from single crowns to full-arch restorations [9,10]. The different Y-TZP types (3Y, 4Y, 5Y) are commercially available and tailored to balance strength and aesthetics [5,11,12]. While 3Y-TZP tends to be more opaque, newer generations such as 4Y-TZP and 5Y-TZP are specifically engineered for higher translucency [13]. However, this increased translucency is accompanied by a reduction in mechanical properties such as flexural strength and fracture toughness [12,14,15].

To broaden clinical indications and meet mechanical and esthetic demands, manufacturers have developed multilayered zirconia materials that combine different Y-TZP types. These multilayered blanks featured gradients in both mechanical strength and optical properties across the disk [11]. Depending on the manufacturer, these strength-gradient multilayered zirconia materials may contain various mixtures and layers within CAD/CAM milling disks, leading to differences in colors, varying levels of translucency, and diverse mechanical properties [14]. While multilayered zirconia materials are increasingly common, a comprehensive understanding of their shrinkage behavior across different material generations and fabrication directions is lacking. This study aims to address this gap using sintering shrinkage analysis.

Manufacturing of zirconia CAD/CAM blanks typically involves uniaxial pressing, cold isostatic pressing (CIP), or hot isostatic pressing (HIP) [11,16,17]. The different variations and mixtures of zirconia powder are filled layer-wise into molds and compressed using above mentioned techniques. After pressing, the “green bodies” are pre-sintered to create porous “white bodies.” This step is crucial to produce stable, millable disks.

To mill restorations from strength-gradient multilayered zirconia using CAD/CAM systems, the shrinkage factor must be considered. It is important to enter the exact batch-specific linear shrinkage into the CAM software. Manufacturers typically measure the linear shrinkage of zirconia for each batch using geometric samples like cubes. This is performed in both the white body and fully sintered states [18]. The shrinkage is calculated as a percentage, and the shrinkage factor is determined by the formula ([volume porous specimen/volume dense sintered specimen] 1/3) [19]. The shrinkage factor is then used during CAD/CAM milling to compensate for the sintering shrinkage. Typical length/linear shrinkages for Y-TZP materials fall in the range of 19–21% for 3-YTZP, with slightly lower values for 4-YTZP and 5-YTZP (approximately 18–19%) [9].

When working with strength-gradient multilayered zirconia, it is important to check whether the shrinkage of 3Y-TZP, 4Y-TZP, and 5Y-TZP within a single disk is consistent. Any variation may affect the fit and clinical outcome of the final restorations. A detailed understanding of these shrinkage properties is essential to ensure dimensional accuracy and long-term success [20,21].

The aim of this in vitro study was to analyze the sintering shrinkage behavior of strength-gradient multilayered zirconia materials and their implications for clinically relevant restorations. Geometrical changes were assessed before and after dense sintering using micrometer screw gauge measurements, digital surface scans, and light microscopy. The null hypotheses tested were that (i) the measurement method, (ii) the vertical position, (iii) the horizontal position within the zirconia disk, and (iv) the type of multilayered zirconia material would have no effect on the shrinkage behavior.

## 2. Materials and Methods

In total, seven zirconia materials were investigated: five strength-gradient multilayered materials composed of 3Y-TZP and 5Y-TZP, and two monolayer single-generation materials (3Y-TZP and 5Y-TZP) serving as control groups. An overview of all materials, including their manufacturers, zirconia type, geometry, color, and batch number, is provided in Table 1.

### 2.1. Specimen Preparation

**Figure 1:**
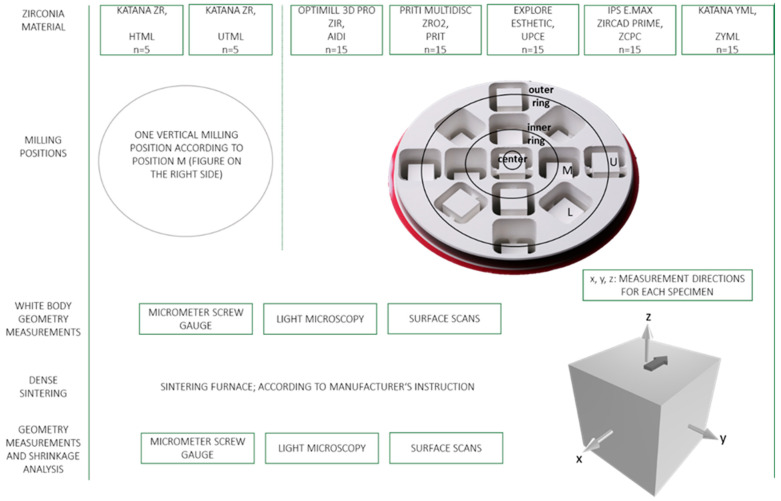
Study design and positioning of specimens within the zirconia disks.

In total, 85 cube-shaped specimens were milled from the seven different zirconia materials (Figure 1). Using CAD software (Ceramill Mind, Version v3.2-9041/64, Amann Girrbach, Koblach, Austria), STL files of a cube of 10 × 10 × 10 mm were positioned within the virtual disk space. For the monolayer control materials (HTML and UTML), specimens were located in the middle vertical position (M, Figure 1). For the multilayer materials, cubes were assigned to three different vertical positions (M = middle; U = upper; L = lower) and three horizontal positions (center; inner ring; outer ring) within the disks. An arrow was positioned on every single cube pointing at the outer rim (see schematic cube in Figure 1), indicating the orientation within the disk. The specimens were milled using a 5-axis CAM unit (Ceramill Motion 2, Amann Girrbach, Koblach, Austria), with one disk used per monolayer material and three disks used per multilayer material per manufacturer. Connecting bars were removed with a laboratory handpiece and rotary instruments. Three varying geometry measurement techniques, described below, were applied for each white body specimen according to three defined measurement directions (x-, y-, z-axis; see schematic cube in Figure 1). The white body specimens were subsequently sintered to a dense state according to the material-specific manufacturer’s instructions in a sintering oven (Mod. LHT 02/16, Nabertherm, Lilienthal, Germany). All specimens were consistently placed at the same height and position within the firing chamber. Moreover, process temperature control rings (Type PTCR-MTH and PTCR-HTH, M.E. Schupp Industriekeramik, Aachen, Germany) were used for each firing to ensure that correct firing parameters are achieved during sintering. The same measurement methods applied for the white body specimens were used for the dense sintered specimens, and the respective length shrinkages were calculated.

### 2.2. Micrometer Screw Gauge

**Figure 2:**
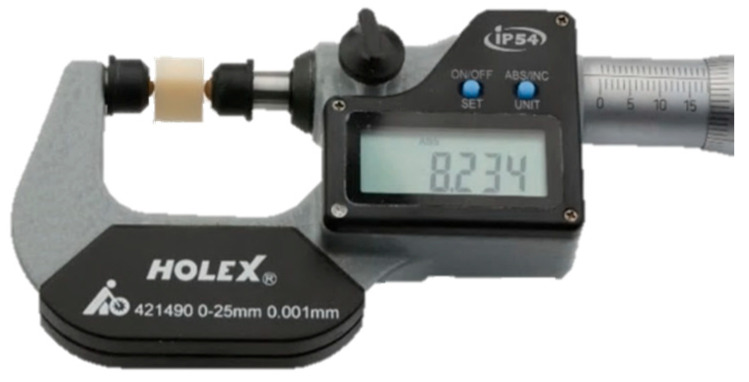
Digital micrometer screw gauge and representative specimen.

Each specimen was measured in the x-, y-, and z-direction (Figure 1) using a calibrated digital micrometer screw gauge (Holex, 421490 0-25, Munich, Germany) with an accuracy of ±0.001 mm (Figure 2). Measurements were conducted both in the pre-sintered (white body) and post-sintered (dense) states.

### 2.3. Light Microscopy

Digital light microscopy (Keyence VHX-970F, Keyence, Osaka, Japan) at 20× magnification was employed to measure each specimen in the x-, y-, and z-directions, both before and after sintering.

### 2.4. Surface Scans

**Figure 3:**
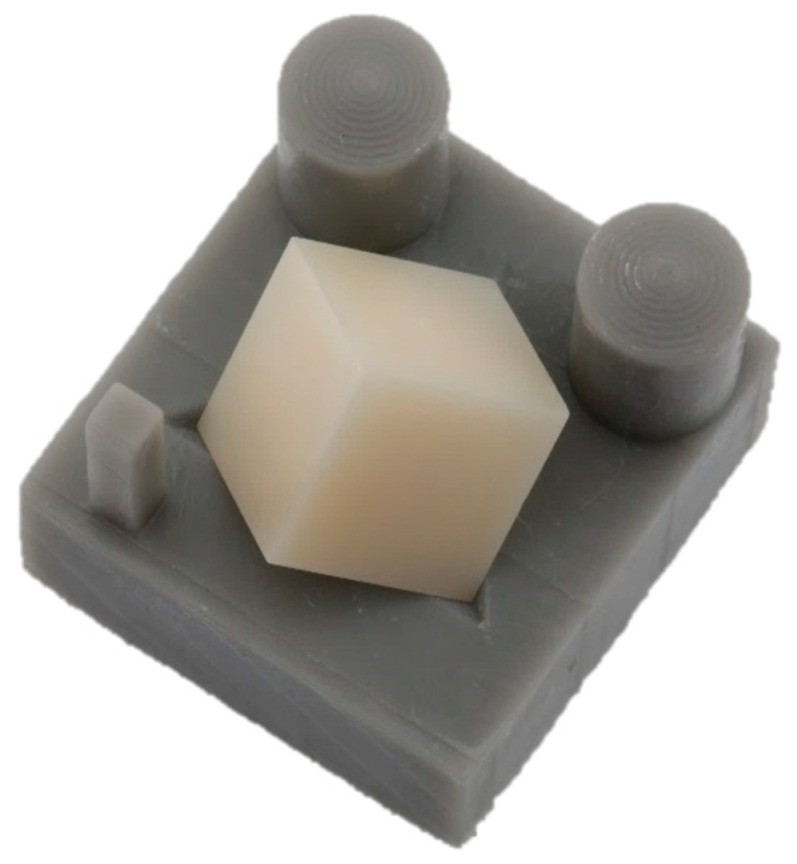
Specimen holder for surface scans.

Surface scans of all specimens were performed pre- and post-sintering using a laser scanner (LAS-20, SD Mechatronik, Feldkirchen-Westerham, Germany) with a resolution of 40 µm in the XY plane. A specific specimen holder was designed to enable simultaneous x-, y-, and z-axis scanning (Figure 3).

**Figure 4:**
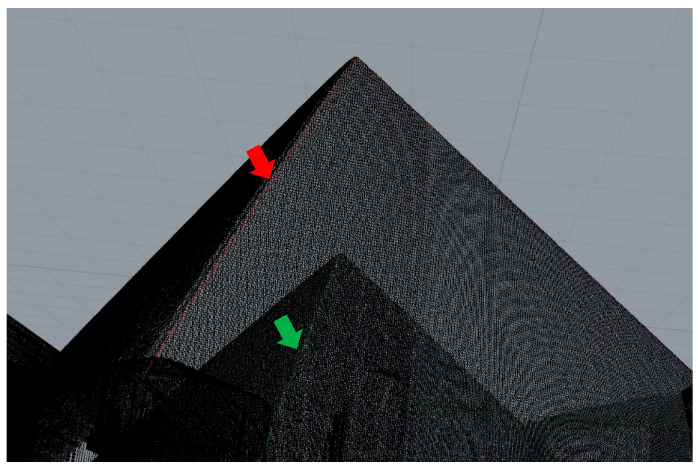
Example of Rhinoceros 3D overlay analysis showing point cloud comparison pre- (red line) and post-sintered (green line).

The resulting point cloud data were analyzed using 3D modeling software (Rhinoceros 3D, Rhino, 7.24, McNeel Robert McNeel & Associates, Seattle, WA, USA). Dimensional changes were determined by axis-specific distance comparisons between pre-sintered and post-sintered point cloud overlays (Figure 4).

### 2.5. Statistical Analysis

All measurements were statistically analyzed using SPSS Version 27.0 (IBM Statistics SPSS 27.0, Armonk, NY, USA). Descriptive statistics (mean and standard deviation) were computed. Normality of distribution was tested via the Kolmogorov–Smirnov test. To determine significant differences, a five-way ANOVA with Scheffé post hoc test and partial eta squared was conducted. Non-parametric Kruskal–Wallis tests with Bonferroni correction were applied to assess the impact of zirconia material and test method within pooled groups. A significance level of *p* < 0.05 was used throughout.

## 3. Results

### 3.1. Global Analysis

The zirconia materials had the highest impact on the shrinkage (partial eta-squared ^η^_P_^2^ = 0.893, *p* < 0.001), followed by the test method used (^η^_P_^2^ = 0.175, *p* < 0.001), while the vertical and horizontal position and measurement direction were not significant (*p* = 0.195–0.763). The effect of the binary, ternary, quaternary, or quinary combinations of the five parameters (zirconia material, test method, vertical position, horizontal position, measurement direction) was significant only for the combinations of zirconia material coupled with test method (^η^_P_^2^ = 0.272, *p* < 0.001), zirconia material coupled with measurement direction (^η^_P_^2^ = 0.111, *p* < 0.001), zirconia material coupled with horizontal position and measurement direction (^η^_P_^2^ = 0.159, *p* < 0.001), zirconia material coupled with test method and measurement direction (^η^_P_^2^ = 0.129, *p* = 0.010), and zirconia material coupled with horizontal position, test method, and measurement direction (^η^_P_^2^ = 0.273, *p* < 0.001) (Table 2).

### 3.2. Impact of Zirconia Materials and Test Methods Within Pooled Data (Vertical, Horizontal Position, and Measurement Direction)

The fixed effects cannot be compared directly, as the higher-order interactions between them were found to be significant. Consequently, several different analyses using pooled data were computed and divided by levels of zirconia material and test method, depending on the hypothesis of interest. The results of the descriptive statistics (mean, SD) are presented in Table 3. Twenty-four percent (5/21) of the tested groups showed deviation from the normal distribution. Data was analyzed non-parametrically.

**Figure 5:**
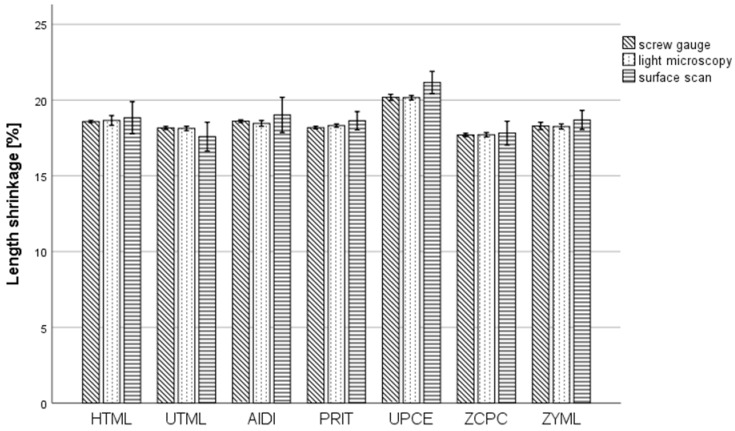
Impact of zirconia materials and test methods within pooled data (vertical, horizontal position, and measurement direction) on length shrinkage.

Within all test methods, UPCE exhibited the highest shrinkage (20.2, 20.1, and 21.1%) among the investigated zirconia materials (*p* < 0.001). Within groups employing micrometer screw gauge and light microscopy measurements, ZCPC showed the lowest shrinkage (17.7%). Comparing the surface scan measurement groups, ZCPC exhibited the lowest values (17.8%) among the multilayer zirconia materials; however, it was in the same range of values (17.6%) with UTML (Table 3, Figure 5).

Within ZYML, UPCE and AIDI the test method surface scan led to higher shrinkage compared to micrometer screw gauge or light microscopy ones (*p* < 0.001). Within PRIT, micrometer screw gauge (18.2%), followed by light microscopy (18.3%) measurements present the lowest and with the test method surface scan (18.6%) the highest shrinkage (*p* < 0.001) (Table 3, Figure 5). Within pooled data, the vertical and horizontal position and measurement direction were not significant (*p* = 0.195–0.763).

### 3.3. Impact of the Horizontal, Vertical Position, and Measurement Point for Each Zirconia Material and Each Method, Separately

Within ZYML and the micrometer screw gauge (*p* < 0.001; y-axis < z-axis < x-axis), as well as light microscopy (*p* < 0.001; z-axis < x-axis = y-axis) test method, the measurement direction showed a significant impact on the shrinkage values (Table 2).

Within UPCE and micrometer screw gauge (*p* = 0.007; middle < upper), the vertical position showed a significant impact on the shrinkage values. Within the surface scan test method, an impact of the measurement direction was observed (*p* = 0.020; y-axis < z-axis). Within AIDI and micrometer screw gauge test method, vertical position (*p* < 0.001; lower < middle = upper), horizontal position (*p* = 0.003; inner ring = outer ring < center), and measurement direction (*p* < 0.001; x-axis = y-axis < z-axis) influenced the shrinkage values. Within ZCPC and surface scan test method, an impact of the measurement direction (*p* < 0.001; x-axis < z-axis) on the shrinkage values was observed.

## 4. Discussion

The present study investigated and compared the shrinkage behavior of monolayer and strength-gradient multilayered zirconia materials consisting of 3Y-TZP, 4Y-TZP, and 5Y-TZP. To the best of the authors’ knowledge, no previous studies have analyzed the shrinkage behavior of strength-gradient multilayered zirconia materials across both vertical and horizontal fabrication directions. Although multilayered zirconia disks are certified and commercially available, practitioners still have concerns about uniform shrinkage and the fit of the final restorations. To address this, dimensional measurements and shrinkage calculations were carried out using methods based on ISO standards and manufacturer practices for determining shrinkage factors in CAD/CAM zirconia disks [19]. This standardized method allows for consistent and comparable results across different materials. It helps to better understand shrinkage behavior in both clinical and laboratory applications.

Among the three measurement techniques applied, surface scanning consistently demonstrated the highest standard deviations across all tested materials. Pairwise comparison revealed that shrinkage values derived from surface scans significantly differed from those obtained using a micrometer screw gauge and light microscopy. However, no significant differences were found between the micrometer screw gauge and light microscopy results. In the present study, the measurements of specimen edge lengths were performed manually, which may have contributed to greater variability due to operator-dependent positioning or alignment inconsistencies. This limitation is likely method-related rather than solely due to specimen preparation. To address this, future studies will focus on automating the specimen positioning and measurement process to minimize user-related variability and improve the reproducibility of surface scan-based evaluations. While three measurement techniques were employed, the study focused on identifying overall shrinkage trends rather than method validation. Each technique was applied consistently, and known limitations such as lower resolution and higher variability of surface scanning were considered in data interpretation. Inter-method calibration and error analysis were beyond the study’s scope but are noted for future research. Consequently, the null hypothesis (i), stating that the measurement method had no impact on shrinkage, was rejected. Regarding material-dependent behavior, the monolayer 3Y-TZP material HTML showed higher shrinkage values (18.6–18.8%) compared to the 5Y-TZP material UTML (17.6–18.2%) (Table 3). These findings align with previous reports indicating decreasing density from 3Y-TZP to 5Y-TZP within multilayer zirconia disks [22].

In strength-gradient multilayered zirconia materials, generations are layered vertically. Based on material composition, it was assumed that lower sections (with higher 3Y-TZP content) would exhibit higher shrinkages than upper sections (with more 5Y-TZP). However, this assumption was not supported by the findings of UPCE and AIDI, where specimens from upper and middle vertical positions exhibited higher shrinkage than those from lower positions. Horizontal specimen location influenced shrinkage only for the AIDI material measured using micrometer screw gauge. Center-positioned specimens shrank more than those from the inner or outer ring, likely due to inhomogeneous green body density caused by pressing during manufacturing. Furthermore, the sintering oven and its temperature distribution can influence the shrinkage behavior. To compensate for this and to ensure comparability between materials, the same number of specimens were always placed at the same height and position within the firing chamber and sintered according to the respective manufacturer’s specifications.

With respect to measurement direction (x-, y-, and z-axes), significant differences were found for ZYML and AIDI when using the micrometer screw gauge (ZYML: y < z < x; AIDI: x = y < z), and for UPCE and ZCPC using surface scans (UPCE: y < z; ZCPC: x < z). In most cases—except for ZYML—shrinkage was higher along the z-axis, indicating a potential anisotropic material behavior during sintering. However, the observed differences remained below 1%, indicating limited clinical relevance.

Thus, null hypotheses (ii) and (iii), which assumed that the vertical and horizontal positioning within the disk would not influence shrinkage, were also rejected. Variability in shrinkage along the vertical and z-direction can be attributed to both the manufacturing process (e.g., uniaxial pressing) and the dominant zirconia type within each layer. Previous studies have similarly found that the fabrication technique influences linear sintering shrinkage in zirconia [16]. The absence of vertical differences in PRIT, ZCPC, and ZYML may reflect uniform blending of zirconia types and layer thicknesses across their disks. In addition to shrinkage behavior, previous research has demonstrated that mechanical properties also vary with the position of restoration within multilayer zirconia disks [23,24]. Thus, both optical and mechanical factors—alongside dimensional stability—should be considered when positioning restorations during the CAD/CAM workflow.

This study focused on linear shrinkage only in standardized cubic specimens, which may limit its clinical relevance. Future research should analyze more complex, clinically representative geometries, such as full-contour crowns and multi-unit bridges, and loading conditions to better reflect functional distortion. Moreover, the lack of phase characterization of the layers and actual layer composition, as well as height within the multilayer disks, can be considered limitations of this study. These parameters should be addressed in future investigations. The resolution of the scanner (XY = 40 µm) used for surface scans should also be noted as a limitation. The vertical resolution affects the accuracy of the point cloud data, and further studies should explore the impact of resolution on shrinkage determination.

From a clinical perspective, the pooled results suggest a largely uniform shrinkage behavior in tested strength-gradient multilayered zirconia materials, supporting their safe and reliable application for CAD/CAM fabricated dental restorations. A non-uniform shrinkage behavior may result in an unfavorable marginal and internal fit of the final restoration. The associated increased cement gap, marginal leakage, or micro-movements under functional loading can be related to restoration failures [25]. The observed uniform shrinkage behavior of tested materials suggests precise internal and marginal fit, supporting long-term restoration performance in clinical practice.

## 5. Conclusions

Within the limitations of this in vitro study, the following conclusions can be drawn:

- The zirconia material itself had the most substantial impact on linear shrinkage, followed by the measurement method applied.
- Among the tested materials, UPCE demonstrated the highest shrinkage values (20.1–21.1%), while ZCPC exhibited the lowest (17.7–17.8%), depending on measurement method.
- The monolayer 3Y-TZP material HTML showed higher shrinkage values (18.6–18.8%) than the monolayer 5Y-TZP material UTML (17.6–18.2%).
- Measurements using a micrometer screw gauge or digital light microscopy resulted in more consistent and less variable shrinkage values compared to surface scan techniques.
- Shrinkage values recorded across the upper, middle, and lower vertical layers of the strength-gradient multilayered zirconia disks did not significantly differ in the global analysis. This supports the assumption of a uniform shrinkage behavior in strength-gradient multilayer zirconia materials for clinically relevant restorations.

## Figures and Tables

**Table 1:** Overview of materials, abbreviations, manufacturers, zirconia type, geometry, color/shade, and batch numbers.

Abbreviation	Material	Manufacturer	Zirconia Type	Geometry d: 98 mm × h:	Color/ Shade	Batch/LOT
HTML	Katana Zr, HTML Plus	Kuraray Noritake (Tokyo, Japan)	3Y-TZP	18 mm	A3	EGLGB
UTML	Katana Zr, UTML	Kuraray Noritake	5Y-TZP	18 mm	A3	EGZJZ
AIDI	Optimill 3D PRO Zir	Aidite (Qinhuangdao, China)	4Y-TZP/ 5Y-TZP	20 mm	A3 standard	26640000
PRIT	Priti Multidisc ZrO_2_ Multicolor	pritidenta (Leinfelden-Echterdingen, Germany)	3Y-TZP/ 5Y-TZP	20 mm	A light, translucent	T0924 AL 20T
UPCE	Explore Esthetic	UPCERA (Shenzhen, China)	4Y-TZP/ 5Y-TZP	20 mm	A3	L2220506019-006 L2211203009-013
ZCPC	IPS e.max ZirCAD Prime	Ivoclar Vivadent (Schaan, Liechtenstein)	3Y-TZP/ 5Y-TZP	20 mm	A3	Z02R88
ZYML	Katana YML	Kuraray Noritake (Tokyo, Japan)	3Y-TZP/ 5Y-TZP	22 mm	A3	EGRKE

**Table 2:** Descriptive statistics of length shrinkage (in %) depending on material, vertical and horizontal position, specimen axis, and measurement method.

Material	Vertical Position	Horizontal Position	Specimen Axis	Length Shrinkage in % (Mean ± SD)		
				Micrometer Screw Gauge	Light Microscopy	Surface Scans
HTML	Middle	Center	x	18.6	18.5	19.7
			y	18.6	18.5	**20.0**
			z	18.5	**18.2**	18.9
		Inner ring	x	18.6 ± 0.00	18.7 ± 0.04	19.2 ± 2.02
			y	18.6 ± 0.00 *	18.8 ± 0.10	18.6 ± 0.73
			z	18.5 ± 0.03	18.5 ± 0.30	17.6 ± 1.64
		Outer ring	x	18.6 ± 0.02	19.2 ± 0.50 *	18.6 ± 0.29 *
			y	18.7 ± 0.06 *	18.7 ± 0.01 *	19.5 ± 0.39
			z	18.5 ± 0.04 *	18.4 ± 0.10	18.4 ± 1.14
UTML	Middle	Center	x	18.1	18.2	17.8
			y	18.2	18.2	16.1
			z	18.1	17.9	**16.0**
		Inner ring	x	18.2 ± 0.02 *	18.1 ± 0.14 *	17.9 ± 0.13 *
			y	18.2 ± 0.04 *	18.2 ± 0.12	18.0 ± 1.29
			z	18.1 ± 0.04 *	17.9 ± 0.09	17.5 ± 1.10
		Outer ring	x	18.3 ± 0.22 *	18.3 ± 0.08	17.9 ± 0.01
			y	18.2 ± 0.03 *	18.2 ± 0.15 *	16.9 ± 0.35 *
			z	18.1 ± 0.10	18.1 ± 0.05	**18.7** ± 0.69
ZYML	Middle	Center	x	18.4	18.0	**17.7**
			y	18.2	18.2	**20.1**
			z	18.1	18.0	18.3
		Inner ring	x	18.6 ± 0.12 *	18.3 ± 0.07 *	19.3 ± 0.85
			y	18.0 ± 0.09	18.4 ± 0.09	18.5 ± 0.73
			z	18.3 ± 0.02	18.1 ± 0.01	19.2 ± 0.38 *
		Outer ring	x	18.2 ± 0.10 *	18.3 ± 0.15	19.1 ± 0.18 *
			y	18.4 ± 0.06	18.7 ± 0.35	18.9 ± 0.84
			z	18.2 ± 0.06 *	18.1 ± 0.03 *	18.7 ± 1.04
	Upper	Center	x	18.8	18.5	18.5
			y	**17.7**	18.2	18.9
			z	18.2	18.1	17.9
		Inner ring	x	18.5 ± 0.05 *	18.3 ± 0.04	19.0 ± 0.47
			y	18.2 ± 0.10 *	18.3 ± 0.03 *	18.6 ± 0.06 *
			z	18.2 ± 0.07	18.1 ± 0.08	18.5 ± 0.07
		Outer ring	x	18.6 ± 0.12 *	18.1 ± 0.05	19.3
			y	18.1 ± 0.01	18.3 ± 0.01 *	17.8
			z	18.2 ± 0.03	18.1 ± 0.26 *	18.2
	Lower	Center	x	18.6	18.1	19.2
			y	18.1	18.3	18.5
			z	18.3	18.1	19.1
		Inner ring	x	18.7 ± 0.27 *	18.4 ± 0.04	18.8 ± 0.22
			y	17.9 ± 0.02	18.3 ± 0.02	18.1 ± 0.38
			z	18.3 ± 0.03	18.3 ± 0.03 *	18.7 ± 0.78
		Outer ring	x	18.6 ± 0.13 *	18.3 ± 0.08 *	19.1 ± 0.21
			y	18.2 ± 0.10	18.3 ± 0.15	**17.7** ± 0.88
			z	18.3 ± 0.03 *	18.2 ± 0.06 *	18.5 ± 0.25 *
PRIT	Middle	Center	x	18.2	18.2	18.4
			y	18.2	18.3	18.7
			z	18.1	18.3	18.2
		Inner ring	x	18.2 ± 0.07	18.2 ± 0.02	**19.5** ± 1.10
			y	18.2 ± 0.05 *	18.2 ± 0.06 *	18.8 ± 0.25 *
			z	18.1 ± 0.08 *	18.4 ± 0.12 *	18.8 ± 0.54 *
		Outer ring	x	18.2 ± 0.02	18.3 ± 0.05	18.2
			y	18.2 ± 0.04 *	18.4 ± 0.16 *	18.6
			z	18.2 ± 0.06 *	18.2 ± 0.05 *	19.4
	Upper	Center	x	18.2	18.2	18.6
			y	18.2	18.2	18.7
			z	18.2	18.2	17.9
		Inner ring	x	18.1 ± 0.01	18.5 ± 0.02 *	19.3 ± 1.01
			y	18.3 ± 0.07	18.3 ± 0.06 *	18.5 ± 0.47
			z	18.1 ± 0.08 *	18.2 ± 0.04 *	18.7 ± 0.99
		Outer ring	x	18.1 ± 0.06	18.4 ± 0.15	18.2 ± 0.27
			y	18.1 ± 0.05 *	18.5 ± 0.02	18.9 ± 0.66
			z	18.1 ± 0.09	18.3 ± 0.02 *	18.3 ± 0.31 *
	Lower	Center	x	18.1	18.5	18.9
			y	18.1	18.4	18.5
			z	18.1	18.4	18.8
		Inner ring	x	18.2 ± 0.00	18.3 ± 0.15 *	**17.8** ± 1.55
			y	18.2 ± 0.10 *	18.3 ± 0.03 *	18.1 ± 0.77
			z	18.2 ± 0.06	18.3 ± 0.15	18.7 ± 0.28
		Outer ring	x	18.2 ± 0.06	18.4 ± 0.04 *	18.6 ± 0.03 *
			y	18.4 ± 0.20	18.3 ± 0.00 *	18.6 ± 0.30
			z	18.2 ± 0.00 *	18.2 ± 0.06 *	19.0 ± 0.57
UPCE	Middle	Center	x	20.2	20.1	21.6
			y	20.2	20.1	20.3
			z	19.1	19.9	21.3
		Inner ring	x	20.2 ± 0.00	20.1 ± 0.00	21.5 ± 0.03
			y	20.3 ± 0.06	20.1 ± 0.06	20.1 ± 0.18 *
			z	20.1 ± 0.04	20.0 ± 0.12	21.3 ± 0.25
		Outer ring	x	20.2 ± 0.00 *	20.0 ± 0.05 *	21.4 ± 0.53
			y	20.2 ± 0.00 *	20.4 ± 0.18 *	20.5 ± 0.47 *
			z	20.1 ± 0.02 *	20.1 ± 0.07	22.5 ± 1.00 *
	Upper	Center	x	20.3	20.1	20.5
			y	20.3	20.3	20.9
			z	20.3	20.1	20.7
		Inner ring	x	20.3 ± 0.04	20.1 ± 0.01	20.8 ± 0.21
			y	20.2 ± 0.01 *	20.3 ± 0.01	21.4 ± 0.50
			z	20.2 ± 0.06	20.2 ± 0.05	**22.3** ± 1.40 *
		Outer ring	x	20.3 ± 0.06	20.2 ± 0.11 *	21.1 ± 0.44
			y	20.3 ± 0.06	20.2 ± 0.03	20.6 ± 0.53 *
			z	20.3 ± 0.28 *	20.1 ± 0.27 *	22.0 ± 0.88
	Lower	Center	x	20.2	**20.0**	21.5
			y	20.2	20.4	21.0
			z	20.2	20.2	20.5
		Inner ring	x	20.1 ± 0.06 *	20.4 ± 0.35	21.1 ± 0.44
			y	20.2 ± 0.08 *	20.2 ± 0.13 *	20.7 ± 0.31 *
			z	20.3 ± 0.51	20.2 ± 0.04	21.2 ± 0.10 *
		Outer ring	x	20.1 ± 0.14	20.2 ± 0.20 *	21.3 ± 1.46
			y	20.1 ± 0.18	20.1 ± 0.02 *	20.7 ± 0.01 *
			z	20.1 ± 0.02 *	20.2 ± 0.07	21.2 ± 0.35 *
AIDI	Middle	Center	x	18.6	18.5	18.1
			y	18.6	18.4	18.2
			z	18.7	18.5	20.7
		Inner ring	x	18.5 ± 0.01	18.5 ± 0.18	18.7 ± 0.58
			y	18.6 ± 0.02	18.4 ± 0.20	18.4 ± 0.10 *
			z	18.6 ± 0.02 *	18.4 ± 0.11	19.1 ± 1.18
		Outer ring	x	18.6 ± 0.04 *	18.1 ± 0.01	19.3 ± 0.89 *
			y	18.6 ± 0.07	18.3 ± 0.13	21.1 ± 1.91
			z	18.6 ± 0.02 *	18.4 ± 0.07	19.7 ± 0.51 *
	Upper	Center	x	18.6	18.6	18.1
			y	18.7	18.4	17.4
			z	18.8	18.5	19.8
		Inner ring	x	18.6 ± 0.08	18.6 ± 0.40	18.7 ± 0.37
			y	18.6 ± 0.04 *	18.5 ± 0.06 *	18.4 ± 1.65
			z	18.7 ± 0.05 *	18.4 ± 0.08	18.7 ± 0.36
		Outer ring	x	18.6 ± 0.02	18.4 ± 0.05 *	19.8 ± 0.50 *
			y	18.6 ± 0.05 *	18.5 ± 0.14 *	19.7 ± 0.52
			z	18.7 ± 0.08	18.5 ± 0.07 *	18.6 ± 2.63
	Lower	Center	x	18.5	18.4	19.5
			y	18.5	18.5	**16.5**
			z	18.8	18.8	**21.6**
		Inner ring	x	18.5 ± 0.03	18.3 ± 0.03	19.1 ± 0.44
			y	18.5 ± 0.06 *	18.3 ± 0.03	19.5 ± 1.87
			z	18.6 ± 0.01 *	18.5 ± 0.27 *	18.7 ± 0.23
		Outer ring	x	18.5 ± 0.02 *	18.6 ± 0.34 *	18.2 ± 0.36
			y	18.5 ± 0.03 *	18.5 ± 0.03	19.0 ± 0.94
			z	18.6 ± 0.04	18.8 ± 0.42	18.6 ± 0.52
ZCPC	Middle	Center	x	17.8	17.5	17.5
			y	17.6	17.7	18.6
			z	17.7	17.7	18.0
		Inner ring	x	17.7 ± 0.06	18.0 ± 0.20 *	17.7 ± 0.55
			y	17.7 ± 0.09 *	17.9 ± 0.05	18.8 ± 0.32
			z	17.6 ± 0.13	17.6 ± 0.03	17.9 ± 0.15
		Outer ring	x	17.6 ± 0.01 *	17.7 ± 0.06	17.1 ± 0.73
			y	17.6 ± 0.04	17.8 ± 0.05 *	17.8 ± 1.42
			z	17.7 ± 0.00	17.6 ± 0.13 *	18.6 ± 0.04
	Upper	Center	x	17.7	17.9	17.7
			y	17.8	17.7	**20.4**
			z	17.7	17.8	17.9
		Inner ring	x	17.7 ± 0.11	17.6 ± 0.00	17.1 ± 0.15
			y	17.6 ± 0.08 *	17.8 ± 0.14 *	17.4 ± 0.99 *
			z	17.8 ± 0.04	17.8 ± 0.20 *	18.3 ± 0.42 *
		Outer ring	x	17.6 ± 0.06	17.7 ± 0.07 *	17.8 ± 0.12 *
			y	17.8 ± 0.28	17.8 ± 0.10	**16.6** ± 0.58 *
			z	17.7 ± 0.14 *	17.6 ± 0.15	17.9 ± 0.90
	Lower	Center	x	17.8	17.9	17.5
			y	17.7	17.6	17.9
			z	17.7	17.8	18.2
		Inner ring	x	17.6 ± 0.06	17.7 ± 0.15 *	17.5 ± 0.61 *
			y	17.8 ± 0.14	17.7 ± 0.00	17.7 ± 0.20
			z	17.6 ± 0.05 *	17.5 ± 0.01 *	18.0 ± 0.63 *
		Outer ring	x	17.7 ± 0.11 *	17.7 ± 0.24 *	17.6 ± 0.10 *
			y	17.7 ± 0.22 *	17.6 ± 0.10	16.8 ± 0.82
			z	17.8 ± 0.24	17.7 ± 0.14	18.6 ± 0.30

* indicates the deviation from the normal distribution. **Bold** values: highest and lowest shrinkage per material.

**Table 3:** Descriptive statistics showing the length shrinkage (in %) for each zirconia material measured using each test method.

Zirconia Material	Micrometer Screw Gauge	Light Microscopy	Surface Scan
	Mean ± SD	Mean ± SD	Mean ± SD
HTML	18.6 ± 0.07 ^deA^	18.7 ± 0.32 ^deA^	18.8 ± 1.06 ^bcA^
UTML	18.2 ± 0.10 *^abA^	18.1 ± 0.15 ^abA^	17.6 ± 0.95 ^abA^
AIDI	18.6 ± 0.09 ^eB^	18.5 ± 0.19 ^cdA^	19.0 ± 1.16 ^cB^
PRIT	18.2 ± 0.08 ^bcA^	18.3 ± 0.11 *^bcB^	18.6 ± 0.61 *^bcC^
UPCE	20.2 ± 0.20 *^fA^	20.1 ± 0.15 ^eA^	21.1 ± 0.73 *^dB^
ZCPC	17.7 ± 0.11 ^aA^	17.7 ± 0.14 ^aA^	17.8 ± 0.79 ^aA^
ZYML	18.3 ± 0.24 ^bcdA^	18.3 ± 0.17 ^bA^	18.7 ± 0.63 ^cB^

* Indicates the deviation from the normal distribution. ^abcdef^ indicates significant differences between the zirconia materials within one test method. ^ABC^ indicates significant differences between the test methods within zirconia material.

## Data Availability

The original contributions presented in this study are included in the article. Further inquiries can be directed to the corresponding author.
